# The Provision of Patient Information about Nystagmus

**DOI:** 10.22599/bioj.105

**Published:** 2018-05-10

**Authors:** Anne Bjerre, Gemma E. Arblaster, Arthur Nye, Helen J. Griffiths

**Affiliations:** 1 Academic Unit of Ophthalmology and Orthoptics, University of Sheffield, UK

**Keywords:** nystagmus, patient information, questionnaire

## Abstract

**Aims:**

The aims of this study were to evaluate the current provision of patient information about nystagmus in orthoptic clinics in the UK and Ireland and to develop a standardised information pack about nystagmus.

**Methods:**

A questionnaire was circulated to orthoptists in the UK and Ireland asking whether they had information to provide to patients with nystagmus, what was included in this information and how it could be improved. Orthoptists were also asked what should be included in a standardised information pack about nystagmus.

**Results:**

Two hundred and thirty three orthoptists completed the questionnaire. One-third of responding orthoptists did not have information to provide to patients with nystagmus. Most reported the information available to them included details of support services and physical symptoms. Including information about living with nystagmus at different ages and long-term prognosis were the most common suggestions to improve information about nystagmus. More than half of orthoptists selected all the suggested topics to be included in a standardised information pack, with support services and long-term prognosis most frequently selected.

**Conclusions:**

Only 67% of responding orthoptists had information about nystagmus to give to patients or their families. Ways to improve the current information and content considered important by orthoptists were taken into account to create a nystagmus information pack, which is now available online.

## Introduction

Information resources for patients aim to provide information relating to diagnosis, symptoms, treatment options, support services, prognosis, daily living, employment and/or education. Information can be provided verbally or in a written format, such as a leaflet or online resource. Information is usually aimed at patients, but also may be for family, friends, carers, teachers or employers. In general practice, patients typically forget 50% of the information they are given by a doctor five minutes after consultation (Kitching 1990), yet when verbal information is supplemented by written or visual stimuli, recall of information from a consultation increases by approximately 30% (Gauld 1981). Written information has been shown to reinforce information discussed verbally and to enable patients to refer back to information discussed in further detail (Dunkelman 1979); it can help patients evaluate advantages and disadvantages of different treatment options, increase patient participation in treatment decisions, help generate realistic expectations (Dey 2004) and increase patient satisfaction (Ley 1988). Without specific patient information, evidence suggests patients then access information from a variety of sources, which may contradict each other, are inconsistent with current research and can result in unrealistic expectations (McIntosh and Shaw 2003).

The provision of collaboratively-produced, evidence-based information from clinicians to patients has been a crucial part of promoting a National Health Service (NHS) predicated on patient empowerment and consumer choice since the 1980s (NHS Executive 1996; Department of Health 1999). Initiatives involving the provision of patient information based on a patient-centred approach that emphasises the priorities and needs of patients rather than an assumption of patient compliance or passivity (Fahrenfort 1987; Dixon-Woods 2001) have been associated with better patient outcomes (Barlow, Turner and Wright 1998; Clark and Nothwehr 1997; Brown 1998; McIntosh and Shaw 2003). The use of patient information has been studied in a variety of healthcare services, including dentistry (Barber et al. 2015), urology (Joshi et al. 2001), breast cancer (Dey 2004), paediatric oncology (James et al. 1988), diabetes (Hardy, O’Brien and Furlong 2001), stroke (Mant et al. 1998) and general practice (Ley 1988). An evaluation of 23 different sources of patient information in dental care found that insufficient information about the disadvantages of treatment was included, leading to a failure to provide sufficient detail for gaining informed consent (Barber et al. 2015). In contrast, a review of 46 articles found that patient information helped breast cancer patients evaluate the advantages and disadvantages of treatment (Dey 2004). A randomised controlled trial of 79 patients who had experienced acute stroke found that patient information did not significantly increase their knowledge of stroke (Mant et al. 1998), yet in patients with low back pain, patient information helped them clarify information from otherwise contradictory sources (McIntosh and Shaw 2003). Ensuring patient information includes comprehensive amounts of information that is balanced and of high quality is therefore an important consideration.

While the use of patient information has been studied in some healthcare services, less research has been carried out into the provision of patient information for ocular conditions, particularly those commonly seen by orthoptists. A randomised controlled trial assessed the impact of information for the parents of children undergoing occlusion therapy for amblyopia. The study found that parents provided with information displayed significantly greater knowledge and concordance with amblyopia treatment (Newsham 2002). The present study evaluated the current provision (in terms of both availability and quality) of information for patients with nystagmus provided by orthoptic departments in the UK and Ireland. Additionally, the study aimed to gain information to help support the development of a standardised information pack about nystagmus.

## Method

Ethical approval for the study was granted by the University of Sheffield Research Ethics Committee (reference number 007481). Prior to inviting orthoptists to complete the questionnaire, an article about the rationale and aims of the project was published in the British and Irish Orthoptic Society’s (BIOS) monthly newsletter, *Parallel Vision*, to raise awareness and maximise the response rate. A questionnaire about the information offered to patients with nystagmus was emailed to NHS orthoptic departments across the UK and Ireland. The questionnaire was generated using Qualtrics software and consisted of five questions. Orthoptists were encouraged to select all the applicable options to ensure complete responses were captured, rather than a forced choice. First, orthoptists were asked if they had information to provide to patients with nystagmus and, if so, in which format the information was delivered (i.e., verbally or written). Those reporting that they had patient information about nystagmus were asked in what way(s) it was useful, if it could be improved and how. Respondents were also asked if they were able to share example nystagmus information with the authors for further evaluation. All orthoptists were asked what information would be useful to include in a standardised information pack about nystagmus. Analysis of the respondents’ data included calculating the number and percentage responses to each question and evaluating free text comments.

## Results

In total, 233 orthoptists responded to the questionnaire. As the anonymous questionnaire was emailed to BIOS members, more than one orthoptist from the same department may have answered the questionnaire. Sixty-seven percent (156 respondents) had information about nystagmus available to give to patients, but 33% (78 respondents) did not. Information was reported to be leaflets (86%), verbal (75%), an information pack (7%) and other (11%). ‘Other’ was described as giving website links and details about the Nystagmus Network charity. One respondent had a nystagmus information board in the waiting room, and one referred to educational services for sensory impairment.

**Figure 1:**
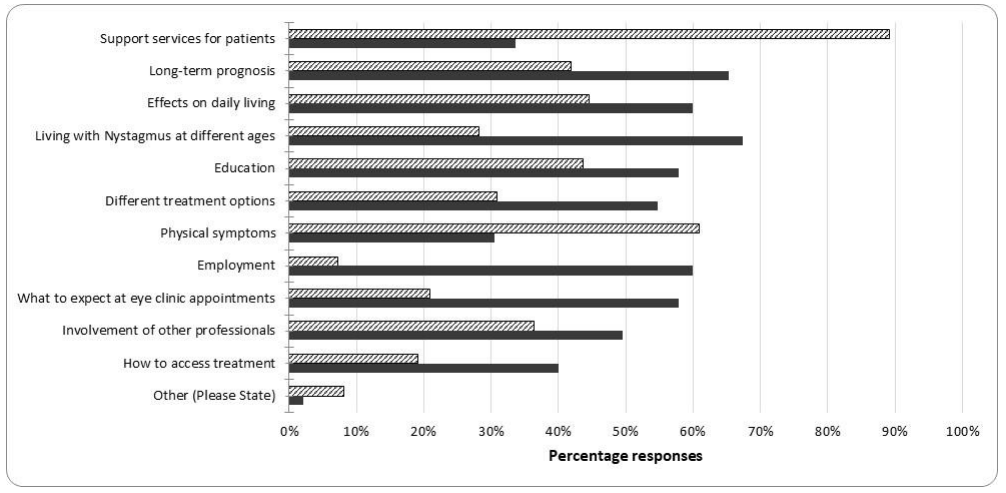
The percentage responses to the questions “In what ways is the leaflet/information pack useful?” (solid black bars) and “In what ways could the leaflet/information pack be improved?” (hatched bars).

One hundred and ten orthoptists responded to the question asking the ways in which their current nystagmus information was useful; percentage responses for each option are shown in Figure 1. Including specific information about support services for patients (89%) and information about physical symptoms (61%) were reported to be the most useful aspects of the current nystagmus information. A smaller proportion felt the leaflet or information pack was useful because it included information about the effects of nystagmus on daily living (45%), education (44%), long-term prognosis (42%) and involvement of other professionals (36%).

Respondents were asked if their current nystagmus information could be improved and in what ways could it be improved. Ninety-five orthoptists responded to this question and percentage responses for each category are also shown in Figure 1. Living with nystagmus (67%), long-term prognosis (65%), effect of nystagmus on daily living (60%) and employment (60%) were reported as areas that could be improved most frequently.

**Figure 2:**
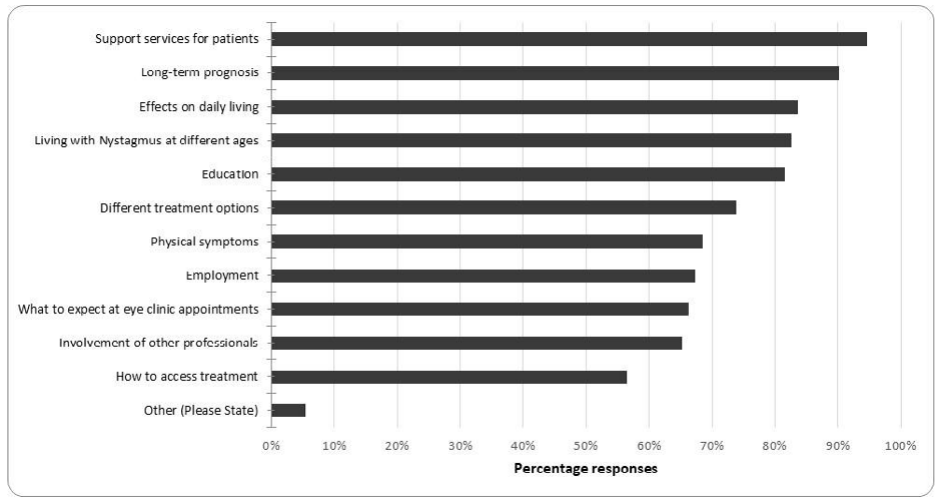
The percentage responses to the question “If standardised nystagmus information was developed, what information would be useful to include?”.

In the free text comment sections many respondents mentioned they used the Nystagmus Network and/or the Royal National Institute for the Blind leaflets and website information, rather than having departmental or hospital-produced written information. To determine what information should be included in a standardised information pack about nystagmus, orthoptists were asked what information was important to include, and the results are shown in Figure 2. Ninety-two orthoptists responded to this question, and all categories were reported as important by more than 50% of respondents. Support services for patients (94%), long-term prognosis (89%), effects of nystagmus on daily living (83%), living with nystagmus at different ages (82%) and education (81%) were most frequently reported as important. Five percent of respondents reported ‘other’ and commented that all categories should be included. No additional information was suggested by respondents.

## Discussion

High quality and available patient information is important in healthcare to help improve patient knowledge, collaborative decision-making, patient satisfaction and patient outcomes (Gauld 1981; Dunkelman 1979; Dey 2004; Ley 1988; Barlow, Turner and Wright 1998; Clark and Nothwehr 1997; Brown 1998; McIntosh and Shaw 2003; Newsham 2002). One-third of orthoptists who responded to the questionnaire did not have specific information about nystagmus to provide to patients. Without high quality information about nystagmus, patients will not receive information that may aid their knowledge of nystagmus (Gauld 1981; Dunkelman 1979), generate realistic understanding and expectations of treatment (Dey 2004) and increase their satisfaction with services (Ley 1988). They may additionally seek information from other sources that are inaccurate or raise unrealistic expectations of their care (McIntosh and Shaw 2003).

Where patient information about nystagmus was available, orthoptists reported the content of the information included some topics, but not all (Figure 1). Eighty-nine percent reported their current information included details of support services; however, other information, such as the physical symptoms of nystagmus and the effects on daily living, were only reported by 61% and 45% of responding orthoptists, respectively. Fewer orthoptists reported that the current information available to give to a patient with nystagmus included information about how to access treatment (19%) and about employment (7%). To improve the current nystagmus information, orthoptists reported they would like to include more information about long-term prognosis (65%), employment (60%), effects on daily living (60%), what to expect at eye clinics (58%), education (58%) and different treatment options (55%). When asked what should be included in standardised information for patients with nystagmus, at least half of all orthoptists selected all the available options. The most frequently selected option was information about support services for patients (94%), while the least selected option was information about how to access treatment (56%). Research into patient information about breast cancer has shown that the type of information included should vary according to patient factors (Dey 2004), which is consistent with the opinions of orthoptists, who responded that information about nystagmus should be relevant to living with the condition at different ages (82%).

### Developing standardised patient information about nystagmus

High quality patient information needs to be valid, relevant, accessible, understandable and accessed at the right time (Dey 2004). As there was an apparent lack of available information about nystagmus that contained all the information orthoptists considered to be important, we have used the results from the study to develop a standardised nystagmus information pack (University of Sheffield Academic Unit of Opthalmology and Orthoptics 2018). It is written in non-medical language (Colledge et al. 2008), but it also includes common terminology. Images have been used to try to increase attention, comprehension, recall and adherence to health instructions, particularly in those with lower literacy (Houts et al. 1998). The nystagmus information pack includes details about infantile and acquired nystagmus. The advantages and disadvantages (Barber et al. 2015) of different management options, with the aim of providing balanced information, is also provided. Patients with a range of different types of nystagmus, their families and orthoptists have been involved in the development of the nystagmus information pack, as patient involvement has been highlighted as important in other areas of healthcare (McIntosh and Shaw 2003). To ensure the nystagmus information pack is accessible (University of Sheffield Academic Unit of Opthalmology and Orthoptics 2018), it is available online, for free, to both patients and clinicians. It also includes other sources of support or information (Houts et al. 1998). It is acknowledged that developing written patient information is not the only factor in improving the information available to patients; communication between clinicians and patients remains crucial (McIntosh and Shaw 2003), and written information should be in addition to this (Dunkelman 1979; Dey 2004; Ley 1988; McIntosh and Shaw 2003).

The limitations of this study include capturing the views only of orthoptists about current information provision and not the views of patients with nystagmus, their families or other professionals involved in their care. Further research evaluating a wider range of opinions may glean more, or different, information than from just one clinical group. Another potential limitation was the survey design, as online questionnaires do not give the opportunity to derive rich, qualitative data in the same way as conversational analysis of interviews or focus groups. Respondents were required to answer questions by selecting from a list pre-determined options, which may not have been exhaustive. Whilst it is possible that important responses were not captured using this method, all survey questions had an option to select “other” and add free text comments. Few comments were added by respondents, suggesting the list of pre-determined options was sufficient to capture their views. Future studies should investigate the impact of patient information, as well as the need for it, and content development. This is particularly important as not all patient information achieves its intended aims, such as increasing patient knowledge and satisfaction (Mant et al. 1998).

To ensure the nystagmus information pack remains up to date, the authors plan to update details, references and website links as appropriate every six months. An evaluation from clinicians’ and service-users’ perspectives of the benefit of this pack will also be conducted.

## Conclusions

In conclusion, the present study showed that only 67% of responding orthoptists had information about nystagmus to give to patients or their families. Whilst the currently available information contained some nystagmus information that orthoptists considered important, there were also many suggestions of how to improve the current information and what to include in a newly created information pack. After consideration of the evidence around patient information from other healthcare professions, a standardised nystagmus information pack has been developed and made available online for free.
